# Development of a novel evaluation method by mathematical anatomy for foot bone alignment displacement using x-ray radiographs

**DOI:** 10.1371/journal.pone.0318556

**Published:** 2025-02-06

**Authors:** Yosuke Maruyama, Katsutoshi Itsukaichi, Naoya Hoshikawa, Takayuki Nakagomi, Tomohiro Matsuyama, Hiroyuki Sasaki

**Affiliations:** 1 School of Rehabilitation, Tokyo Professional University of Health Sciences, Tokyo, Japan; 2 Institute for Advanced Medical Sciences, Hyogo Medical University, Nishinomiya, Hyogo, Japan; 3 Department of Rehabilitation, Faculty of Health Care and Medical Sports, Teikyo Heisei University, Ichihara, Chiba, Japan; 4 Department of Orthopaedic Surgery, Kamiya Hospital, Tokyo, Japan; 5 Department of Therapeutic Progress in Brain Diseases, Hyogo Medical University, Nishinomiya, Hyogo, Japan; Mustansiriyah University, IRAQ

## Abstract

The major methods of evaluating the foot arch in clinical practice in patients with foot deformities are medial longitudinal arch measurement using body surface somatometry and radiographic morphometry. Although these methods are widely used, they are considered problematic in terms of differences in scores between the methods. In this study, we developed a new geometric shape analysis method for the bony arrangement of the foot using the two-dimensional fast Fourier transform (2D-FFT), which incorporates mathematical anatomy using x-ray radiographs. Lateral radiographs of the foot bones were obtained using ImageJ2 provided by the National Institutes of Health. The 2D-FFT images show the characteristic directional power spectrum extending from low to high frequencies in the first and third quadrants on the normal, low and high arched foot respectively. The current method of reflecting the bone arrangement status of the metatarsal and tarsal bones may have the potential to establish a new radiographic evaluation method for assessing abnormal foot–bone alignment. As a result, the foot bone 2D-FFT method can be useful in assessing the medial longitudinal arch and predicting the prognosis of these patients.

## Introduction

The foot’s main functions are to support body weight and absorb impact from the ground. The complex bony arrangement of the foot makes it difficult to understand its structural and functional status, with many bones articulating to achieve various motions. The arch of the foot can be divided into distinct arches. The longitudinal arch includes the medial and lateral longitudinal arches, whereas the transverse arch exists and functions at the tarsal and metatarsal levels. The height of the medial longitudinal arch is measured by several methods to categorize the foot structure as low arch (pes planus), normal arch (pes rectus), and high arch (pes cavus). The causes of these foot deformities vary, ranging from congenital to acquired, due to development, disease, and lifestyle. They also manifest from various causes [1–3]. In addition, low-arched feet have been reported to cause various functional impairments, such as weakness of the plantar muscles and decreased balance and walking ability [4]; thus, it is imperative to understand the status of the foot arches.

There are two major methods of evaluating the medial longitudinal arch. One is the body surface somatometry, such as the arch height ratio, arch index, footprint, or foot posture index-6, measured from the body surface. The other is the radiographic morphometry, such as the calcaneal pitch angle, lateral talo-first metatarsal angle (Méary’s angle), calcaneo-first metatarsal angle (Hibbs angle), and navicular index, which measure the angle and distance of bone indices from radiographs [5–7]. The two evaluation methods are often used: the body surface somatometry is often used as a quick and simple evaluation method, while the radiographic morphometry is often used in medical situations because it allows more detailed observation, despite the risk of radiation exposure. There have been many studies on the relationship, validity, and reliability of body surface somatometry and radiographic morphometry, and our team has reported variations among the evaluation methods in terms of correlation [8–16]. However, there were risks of errors in the body surface and radiographic image evaluation methods due to the degree of skill in palpation techniques, the influence of soft tissues, and errors in the measurement points of bone indices, respectively [17, 18]. In addition, differences among evaluation methods remain though cutoff values exist for each evaluation method.

Recently, new evaluation methods were developed [19–21]. However, owing to the complexity of the foot arch structure, each evaluation method has its advantages and disadvantages, and there is still no uniformity in clinical practice and research fields. Under these circumstances, algorithms to computationally represent various biological structures have been developed. The field of mathematical anatomy was created, playing a major role in diagnostic imaging and surgery besides anatomy [22]. In this study, we attempt to develop and establish a new geometrical shape analysis method for the bony arrangement of the foot using the two-dimensional fast Fourier transform (2D-FFT), which is a certain method of these mathematical anatomy. A Fourier transform is a transformation that maps a complex or real function *f* of a real variable to another function of the same type *F*. In general, it is a process or formula for mapping a function or signal to an analysis in terms of its frequency, and can be said to be a method of decomposing a function *f* into sine and cosine waves. On the other hand, the transform that returns *f* is called the inverse Fourier transform. As Fourier analysis is useful to evaluate the image based on the different biological greyscale images, this method has been used for biological texture analysis and can provide a quantitative assessment of macro- or microstructures, which will help to elucidate structural properties [23, 24].

We performed 2D-FFT as a spatial frequency analysis of 2D lateral foot x-ray radiographs of the medial longitudinal arch, which shows a complex three-dimensional (3D) structure, to establish the foundation for a rapid, reproducible, accurate, and objective analysis method. This study used the ImageJ2 image analysis software released by the National Institutes of Health. It is highly versatile since it can run on various operating systems, such as Microsoft Windows, Macintosh, and Linux, and it is free since it is open source and has the latest global algorithms created in academic research as plug-ins [25].

Quantitative assessment of macro or microstructures, such as shape, relative foot bone volume, foot bone spacing, and connectivity, may improve the dissimilarity of bone arrangements. This study is expected to provide a new conceptual evaluation method for radiographic morphometry to observe the state of the medial longitudinal arch, a phenotype of abnormal tarsal alignment, with less human intervention through a simpler reference point setting compared with conventional methods. In addition, the temporal analysis of the foot bone alignment changes from onset to recovery in patients with foot dysfunction caused by diseases resulting in foot deformity should be applied clinically to understand, evaluate, and predict the prognosis of the patients’ conditions.

## Subjects and methods

The study was approved by the ethical committee of the Tokyo Professional University of Health Sciences (Nos.: TPU-21-011 and TPU-23-019) and conducted in accordance with the Ethical Guidelines for Clinical Studies in Japan, published by the Ministry of Health, Labour and Welfare, and the principles expressed in the Declaration of Helsinki. Written informed consent was obtained from all patients. The patients for this study were recruited from August 10th 2021 to March 29th 2024. All data underlying the findings presented in this report will be made available without restriction from the corresponding author upon reasonable request.

### Subjects

Of 69 adults enrolled in this study, we excluded those with any disease, injury, or history of disease in the lower extremities, resulting in a final sample of 60 healthy adults (38 males, 22 females; age: 25.2±6.4 years; height: 166.8±8.5 cm; weight: 61.6±12.3 kg; body mass index: 22.2±2.9 kg/m^2^). Subjects were classified into three groups according to the arch height ratio [26] in the bipedal standing position, a method frequently used in clinical practice: low arch group for arch height ratio <15, normal arch group for arch height ratio 15–20, and high arch group for arch height ratio >20 (Table 1).

**Table 1:** Classification of subjects with arch high ratio.

	Normal arch (N = 38)	Low arch (N = 11)	High arch (N = 11)
Mean±SD	17.81±1.35	13.78±0.80	21.69±1.53
Maximum	19.78	14.81	25.00
Minimum	15.26	11.84	20.09

Values are presented as means ± SDs. SD, Standard deviation.

### Methods

#### Radiographs

Digital radiographs were obtained barefoot in a natural one-leg standing loading position with no specific instructions using Radiographic System REF Radnext 50 at a tube voltage of 50 kV and tube current of 100 mA (FUJIFILM Healthcare Corp., Tokyo, Japan). For lateral radiographs, the point of incidence was placed on the talus bone, the distance was 100 cm, the irradiation time was 0.2 s, and the irradiation range was the minimum to cover the entire foot area. During image recording, each subject wore a protector to reduce radiation exposure. The measurement software, PDI Viewer Version 2.20 R03 (Konica Minolta, Inc., Tokyo, Japan), was used to calculate the lateral first metatarsal tilt angles.

#### 2D-FFT analysis

**Fig 1:**
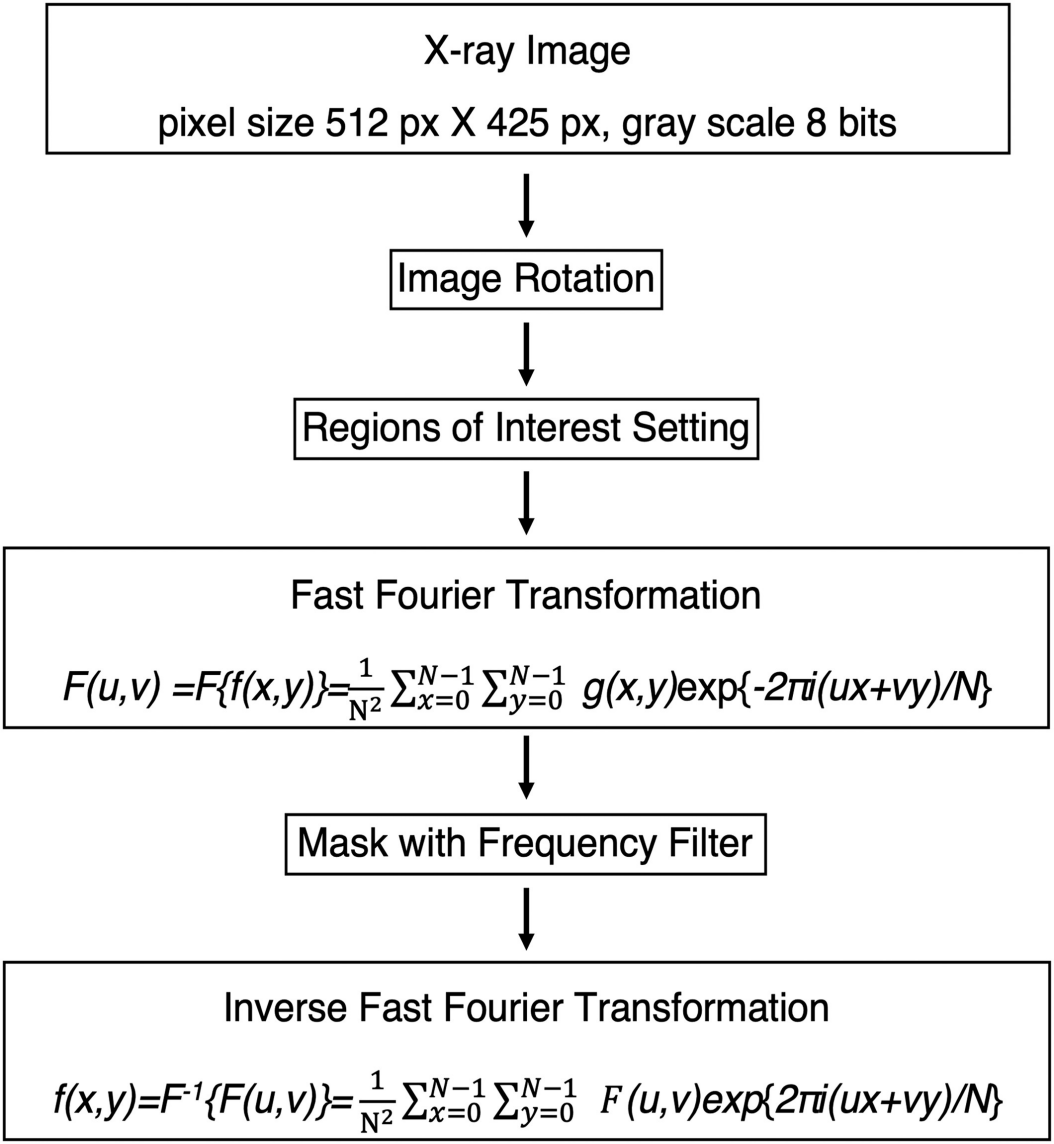
Overall scheme of the texture analysis of lateral foot bone. The x-ray radiographs were leveled, and then regions of interest were set up with the upper surface of the talus pulley as the upper edge, the bottom from the calcaneus base to the metatarsal head base, and the right and left edges to the calcaneal prominence and metatarsal head. The 2D-FFT and Inv-FFT [see Refs. 22 and 23] in these regions of interest were processed using ImageJ2 image processing software (Ver. 2.9.0/1.53t, National Institutes of Health, Washington, D.C., USA, https://imagej.nih.gov/ij/).

An overview of the analysis procedure for the radiographs is shown in Fig 1. For the implementation of this method, an Apple iMac (Intel i7, 4 GHz, 32 GB memory) and Panasonic CF-LV (Windows10 Intel i7, 1.9 GHz,16 GB memory) were used. The original 8-bit images of 512×425 pixels were processed with the ImageJ2 image processing software (Ver. 2.9.0/1.53t, National Institutes of Health, Washington, D.C., USA, https://imagej.nih.gov/ij/).

1. Leveling: Grid lines were displayed in the radiographs, and image rotations were adjusted so that the line connecting the calcaneus base and the metatarsal head base was horizontal using the rotate command of ImageJ2.
2. Reign of interest (ROI) setting: To limit the analysis to the medial longitudinal arch, each analysis area was defined as the upper surface of the talus pulley at the upper end, the basal surface of the calcaneus to the basal surface of the metatarsal head at the lower end, and the left end from the calcaneal prominence to the metatarsal head at the right end, respectively, and the ROI was set to remove areas outside the area.
3. 2D-FFT: FFT processings were performed in the ROI using the FFT command. The ROI images were reconstructed by inverse fast Fourier transform (Inv-FFT) using its command.
4. The obtained 2D-FFT images (512 x 512 pixels; 8-bit; 256K) were analyzed using ImageJ2’s superimposition, angle measurement, and binarization commands.

## Results

The analysis method focused on the medial longitudinal arch, and ROIs were defined from lateral foot x-ray radiographs. 2D-FFTs were performed on the black-and-white shades of gray that indicated the positional relationship of each foot bone (Fig 1). The 2D-FFT program is as follows. Briefly, the gray level of each image pixel was represented by a function, *f(x*,*y)*, where *x* and *y* are the Cartesian co-ordinates of a pixel point. The 2D-FFT of *f(x*,*y)* is written *F(u*,*v)* and is described by Eq (1).


$$F\left(u,v\right)=F\left\{f\left(x,y\right)\right\}=\frac{1}{{\text{N}}^{2}}\sum_{x=0}^{N-1}\sum_{y=0}^{N-1}\;f\left(x,y\right)\text{exp}\{-2\pi i\left(ux+vy\right)/N\}$$ (1)

Inv-FFT is written *f(x*, *y)* and is described by Eq (2).

$$f\left(x,y\right)=F^{-1}\left\{\;F\left(u,v\right)\right\}=\;\frac{1}{{\text{N}}^{2}}\sum_{x=0}^{N-1}\sum_{y=0}^{N-1}\;F\left(u,v\right)exp\{2\pi i\left(ux+vy\right)/N\}$$ (2)

where *u* and *v* are spatial frequencies corresponding to the *x*- and *y*-axis of the original image, respectively. The value for N is surface integral, *dxdy*.

**Fig 2:**
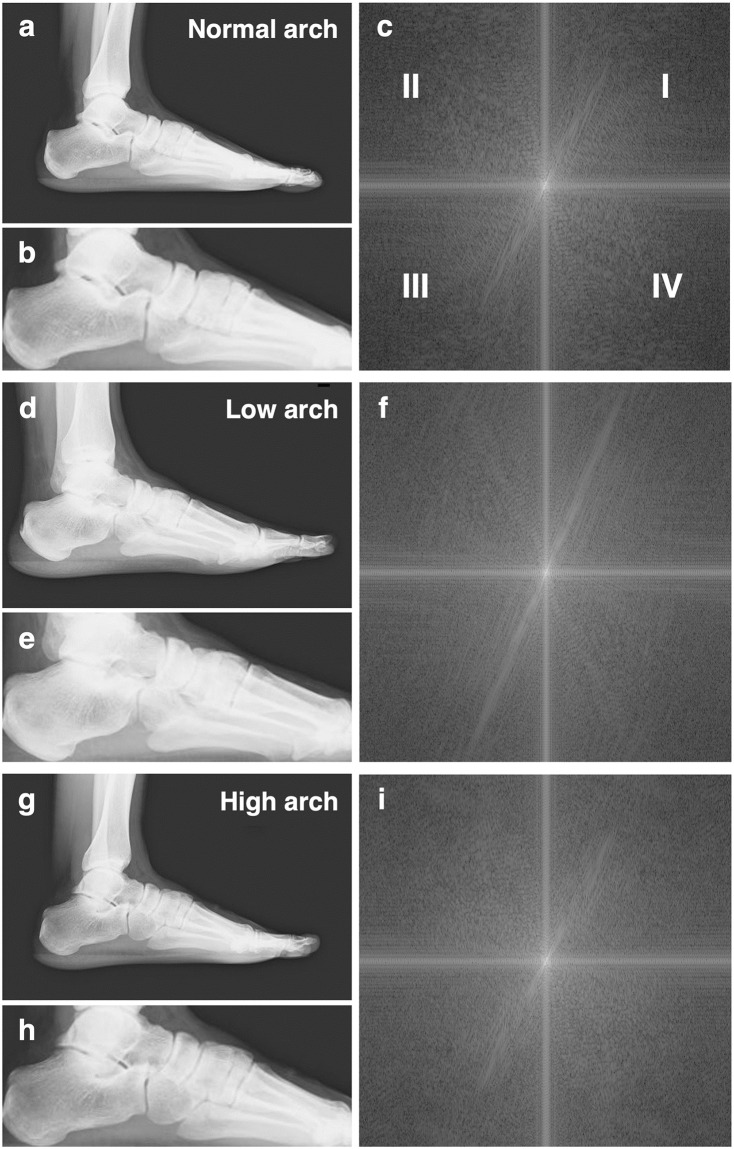
The texture analysis of lateral foot bone. The analysis process for a normal arch foot (a-c), a low arch foot (d-f), and a high arch foot (g-i) in each example is shown below. The original x-ray radiographs (a, d, and g) were leveled. Then ROIs were set up with the upper surface of the talus pulley as the upper edge, the bottom from the calcaneus base to the metatarsal head base, and the right and left edges to the calcaneal prominence and metatarsal head (b, e, and h). The 2D-FFTs in these ROIs were processed using ImageJ2 (c, f, and i). Roman numerals I, II, III, and IV indicate the respective quadrants.

**Fig 3:**
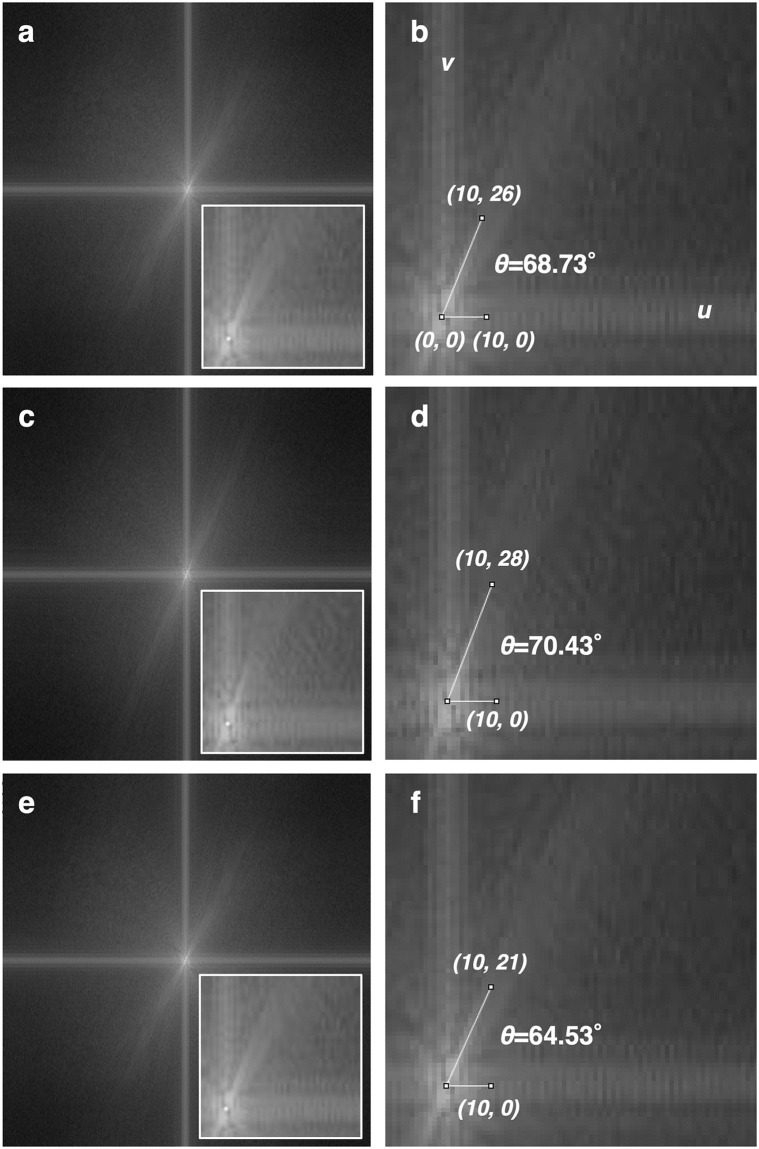
Angular measurement of the power spectrum of the normal arch group, low arch group, and high arch group. (a and b) Normal arches, (c and d) low arches, and (e and f) high arches. All insets of a, c, and e were high-power views of the first quadrant. All power spectrum images obtained from 38 cases in the normal arch group (a), 11 cases in the low arch group (c), and 11 cases in the high arch group (e) were superimposed within each group to obtain an average power spectrum image. The angles (*θ*) in the low-frequency region for the high-intensity regions seen in this power spectrum were 68.73° (b) in the normal arch group, 70.43° and (d) in the low arch group, and 64.53° (f) in the high arch group. (*u* = ±256, *v* = ±256).

Sixty subjects were classified into three groups according to arch height ratio, resulting in 38 cases in the normal arch group, 11 cases in the low arch group, and 11 cases in the high arch group (Table 1). All radiographs in normal, low, and high arch groups were leveled (Fig 2a, 2b, 2d, 2e, 2g and 2h). Then the power spectra were obtained by foot bone 2D-FFT calculation in ROIs radiographs. In the resulting power spectra, all three groups contained a constant directional component extending to high frequencies in the first and third quadrants (Fig 2c, 2f and 2i). All power spectra were superimposed in 38 cases in the normal, 11 cases in the low, and 11 cases in the high arch groups, respectively, to obtain the mean value of the spectra in each group (Fig 3a, 3c and 3e). The power spectral population with constant directionality in the three groups showed different slopes in the high-frequency region within *u* = ±15 between the groups (Fig 3a, 3c and 3e insets). The base point on the u-axis was set at *u* = 10, from which luminance measurements were taken on a perpendicular line along the v-axis, and the point with the highest luminance was set at *v*, as described in Eq (3).


$$\theta \;={\text{tan}}^{-1}\left(\frac{v}{u}\right)$$ (3)

In the normal group, the spectral population function was *u* = 10, *v* = 26 with angle *θ* = 68.73°, whereas the low arch group had *u* = 10, *v* = 28 with angle *θ* = 70.43°, and the high arch group had *u* = 10, *v* = 21 with *θ* = 64.53° (Fig 3b, 3d and 3f).

**Fig 4:**
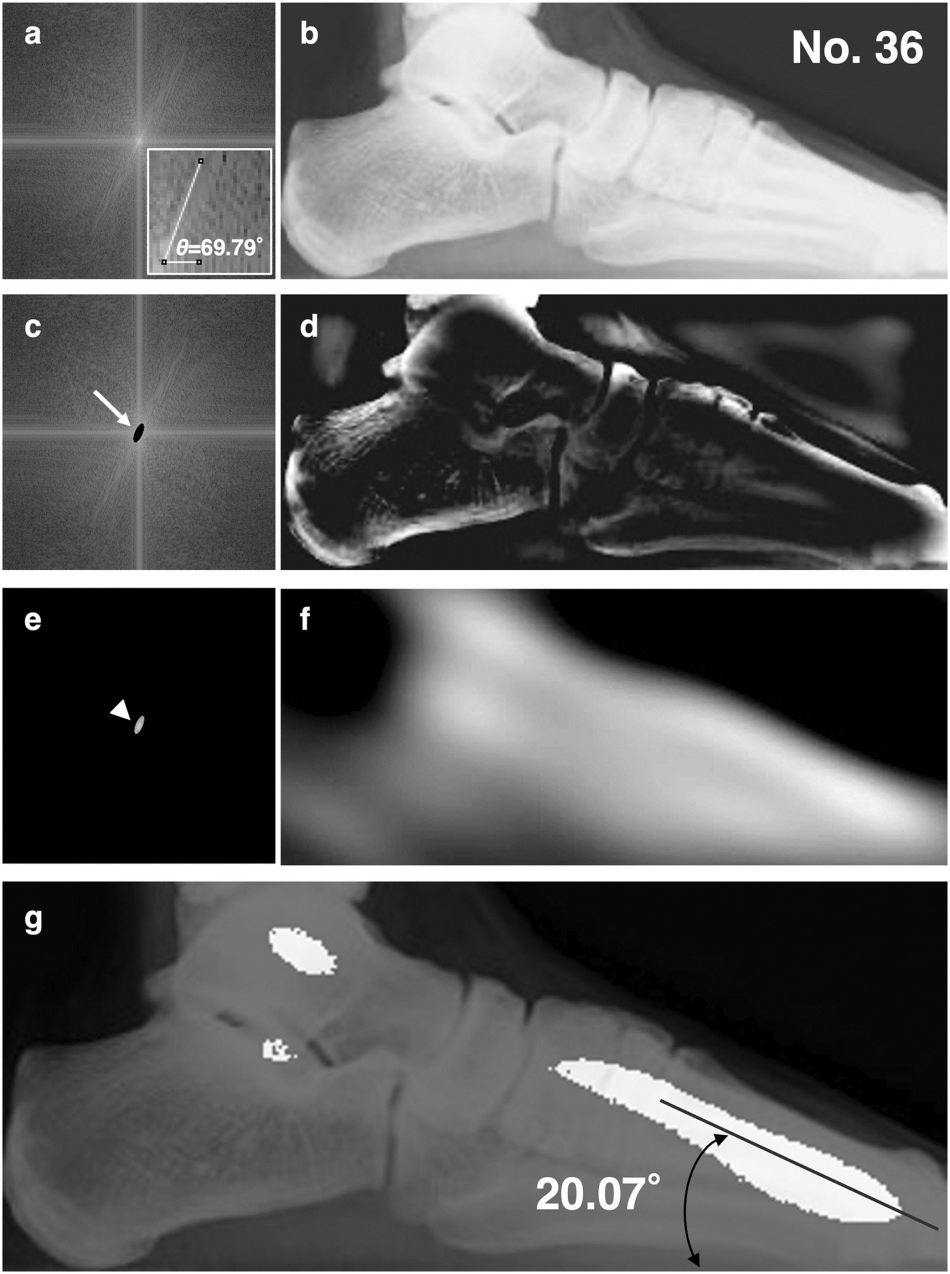
Inv-FFT images of the high-frequency and low-frequency regions. The images were reproduced using a high-pass or low-pass filter on a No.36 subject with a normal arch. (a) A whole 2D-FFT image of the subject. Inset, high-power views of the first quadrant with *θ* = 69.79° of power spectrum angle. (b) Inv-FFT image obtained from (a). (c) A high pass filter (arrow) and (d) Inv-FFT image from (c). (e) A low pass filter and (f) Inv-FFT image from (e). The unfiltered area is indicated by an arrowhead. (g) Inv-FFT image in the low-frequency domain superimposed with the original x-ray radiographic image. The inv-FFT image in the low-frequency domain was overlaid on the original image as a binarized image with adjusted threshold, and was found to be consistent with the metatarsal with a tilt angle of 20.07°.

To further interpret the directional components, we attempted an Inv-FFT of the low and high frequency components of sample #36 with a typical normal arch extending from high to low with a first metatarsal tilt angle of 20.07° (Fig 4). An ellipsoid-shaped filter was set up in the FFT image with major axes *u* and *v* as (+14, +38) and (-14, -38) and minor axes *u* and *v* as (+6, -16) and (-6, +16) covering the high intensity spectrum extending to the first and third quadrants. The inner and outer sides of this ellipse are the high-frequency and low-frequency sides, respectively. We found that the Inv-FFT of the low-frequency component with a spectral base angle *θ* = 69.79° indicated a spatial arrangement of the foot bones (Fig 4a and 4b). Furthermore, the Inv-FFT of the high-frequency component indicated the internal bone trabeculae and bone contour (Fig 4c and 4d). When the threshold of the image obtained by Inv-FFT of the low-frequency component was varied and the binarized image was superimposed on the original radiographic image (Fig 4e and 4f), it was consistent with the first metatarsal (Fig 4g). Therefore, the tilt angle of the first metatarsal was assumed to have a similar tilt tendency to the orthogonal angle *Φ* = 90°–*θ* of the power spectral population obtained from the 2D-FFT.

The lateral first metatarsal tilt angles of the ROI set radiographs and the orthogonal angles *Φ* were measured in the normal, low, and high arch groups, and the mean values of each group were calculated using the PDI Viewer measurement software. The normal group was 18.35±2.49°, the low arch group was 16.54±1.62°, and the high arch group was 21.13±2.82° (Table 2). On the other hand, the orthogonal angle *Φ* was 21.21 ± 2.46° in the normal group, 18.76 ± 2.39° in the low arch group, and 25.07 ± 2.76° in the high arch group.

**Table 2:** Comparison of arch height ratios, lateral metatarsal tilt angles and orthogonal angles of power spectral populations derived from 2D-FFT.

		Arch-height ratio	Lateral first-metatarsal tilt angle	*Φ* (= 90-*θ*)
Normal arch (N = 38)	Mean±SD	17.81±1.35	18.35±2.49°	21.21±2.46°
	Maximum	19.78	22.60°	26.56°
	Minimum	15.26	12.70°	17.35°
Low arch (N = 11)	Mean±SD	13.78±0.80	16.54±1.62°	18.76±2.39°
	Maximum	14.81	18.80°	24.04°
	Minimum	11.84	14.00°	15.18°
High arch (N = 11)	Mean±SD	21.69±1.53	21.13±2.82°	25.07±2.76°
	Maximum	25.00	25.40°	31.58°
	Minimum	20.09	17.10°	21.69°

Values were calculated from 38 cases in the normal arch group, 11 cases in the low arch group, and 11 cases in the high arch group. *Φ* is the orthogonal angle of the superimposed power spectral population (*θ)* derived from the 2D-FFT in the low arch group, the normal arch group, and the high arch group, respectively. Values are presented as means ± SDs. SD, Standard deviation.

## Discussions

Each living body component has unique characteristics, such as shape, arrangement, color, and condition of the material surface. In this study, we analyzed the characteristics of the constituent elements using image analysis techniques by devising a method for extracting elements from images using values that indicated the characteristics and verified the method’s effectiveness. There are many methods in bio-medical image processing for extracting and analyzing the features of radiographic images [22]. Of these, texture analyses are methods for quantifying the conditions that appear on the surface layer of a material as a function of spatial variation in pixel intensity and are generally classified into structural and statistical methods. The 2D-FFT analysis method used in this study is included in the statistical method [23, 24].

The foot bone 2D-FFT uses lateral foot radiographs and ROIs at the metatarsals and tarsals that form the foot arch, excluding the phalanges. It is established that this is a new mathematical evaluation method for geometric analysis using the power spectrum obtained by 2D-FFT. Even in the three groups classified with the arch height ratio [26] which contain possibilities of some errors due to bone index settings and measurement values, the foot bone 2D-FFT proposed in this study can reflect the arrangement of metatarsal and tarsal bones forming the arch and is a simple, rapid, accurate, reproducible, and objective evaluation method.

In this study, the foot bone 2D-FFT revealed high-intensity power spectrum populations with a constant direction through the origin from the first (+*u*, +*v*) to the third (-*u*, -*v*) quadrant of the coordinates, and the normal arch, low arch, and high arch groups showed differences in the angles of the low-frequency spectra. Hence, the low arch group had power spectral populations closer to the *v*-axis, the high arch group had lower angles, and the normal arch group fell between the oblique lines of the low and high arch groups, suggesting that the difference in angles resulted from the degree of formation of the longitudinal arches of the foot. To confirm the hypothesis, several Inv-FFTs were performed on a 2D-FFT image using low-pass and high-pass filters, which depicted a short columnar image reflecting bone in the first metatarsal and talus in the image from the low-frequency component at the origin. The foot bone 2D-FFT power spectrum can be estimated to represent the tilt of the first metatarsal bone since the binarized Inv-FFT image overlaps with the positions of the first metatarsal bone when the original ROI-set radiographs are superimposed. The metatarsal angles from the radiographs and the angles calculated from the tarsal FFT showed a similar trend in all three groups (Table 2). In contrast, some differences were observed between the measured and arithmetic values. Further validation with more cases will be required in the future.

In the case of foot bone 2D-FFT, besides the first metatarsal, medial cuneiform, navicular, and calcaneus bones that constitute the medial longitudinal arch are included in the ROI and their arrangement characteristics. In our recent report, the correlation between radiographic morphometry and body surface somatometry for the medial longitudinal and transverse arches showed a slight correlation due to the difficulty of setting bone indices in the radiographs, errors in the somatometric evaluation method due to excess soft tissue, and most significantly, the complex relationship between the foot arch structure and the 3D arrangement of tarsal bones, which contain many bones and joints [16]. Similarly, a few reports show no relationship between longitudinal and transverse arches [27, 28]. In this study, mathematical anatomy analysis using 2D-FFT of the foot bones, which show complex 3D structures, could be simplified to the tilt of the first metatarsal bone. Further studies will establish the benefit of the foot bone 2D-FFT, which covers all tarsal bones constituting the foot arch including the lateral longitudinal, medial longitudinal and transverse arches, as a comprehensive evaluation method covering the morphological changes of all the foot arches besides being an evaluation method for the medial longitudinal arch. In addition, it can elucidate the cause and/or functional mechanism of the high and low arches of the foot. In this study, we hypothesized mathematical heterogeneity in foot deformity using a 2D-FFT analysis. We expect to obtain more valuable results in the future by increasing the number of cases, clarifying in more detail what the power spectra shown in the 2D-FFT images of the footbones indicate, and providing a solid rationale for our results. This analysis method requires very little intervention compared to the conventional analysis method involving several manual measurements because the only reference is the angle measurement. Therefore, the margin of error is considered small. The angle measurement is also accurate because it is performed using ImageJ. We are currently conducting research to clarify the relationship with other evaluation methods and the inter- and intra-rater reliability and validity of the newly proposed evaluation method. After confirming inter- and intra-assessor reliability, we aim to verify and make observations under various conditions, such as differences in age, sex, bone density, and disease cases. In addition, it may help to predict tibialis posterior tendon dysfunction based on abnormal tarsal bone alignment in foot dysfunction caused by diseases that will lead to abnormal foot deformities in the future [29], validate the efficacy of flat foot surgery, and understand various conditions with high accuracy. Furthermore, in conditions where muscle tone changes due to central or peripheral nerve damage, such as cerebrovascular disease, it is expected that temporal analysis of changes in foot bone alignment from onset to recovery will be helpful in patient assessment and prognosis prediction.
